# Prof. Wright and Operative Dentistry

**Published:** 1872-01

**Authors:** W. E. Driscoll


### PROF. WRIGHT AND OPERATIVE DENTISTRY.

BY W. E. DRISCOLL.

In the DENTAL REGISTER of October, 1871, is a paper from the pen of Prof. C. M. Wright, on Operative Dentistry.

The Professor's conclusions, where supported by the necessary facts, are excellent. But if some of his deductions are not without this recommendation to our serious consideration, it certainly devolves upon him yet to prove it. He says: "At the meeting of the American Dental Association, at Nashville, Dr. Forbes excused himself for not being able to make himself heard, on the ground of having lost his teeth by 'first-class dentistry.' Thousands of mouths are in like condition." No dentist worthy the name needs be told that the assertion that thousands of mouths are toothless from "first-class dentistry" is utterly reckless, both of facts and the consequences of such mischievous teaching. If true, it would brand the talented and educated of the profession as a band of charlatans that should be suppressed and punished by law.

The worst effect of such declarations is to throw the weight of what influence the name of the writer may have against the elevation of those who are doing so much to degrade our specialty from its just rank among the other professions.

It may, also, as the writer seems to expect, lower his own standing in the estimation of those who are laboring to make our calling honorable. Were such a charge true, he would owe it to humanity to prove its truth; but, instead, he contents himself with the naked assertion. Since committing himself to such an extent, he owes it to himself, also, to produce some evidence to justify such a charge.

Further on Prof. W. says: "A great many men, who have come into the profession within ten years, in our State, have never seen a soft foil filling, and do not know the advantages." We only notice this to say that, if true, it is a subject well worth the attention he calls to it. A dentist that does not know the advantages of soft foil, when indicated, has indeed much to learn, just as those "elegant operators throughout the South" have, "who use soft or non-adhesive gold entirely;" either of which may be aptly compared with other doctors who have *one* remedy for all stages or varieties of a given disease.

But our essayist is dissatisfied because so much more is being said of adhesive foil than soft foil.

It occurred to us, sometime ago, that the extra amount of attention adhesive foil was attracting at present, was because it was taken as granted that everybody knew how to fill such cavities as were suitable for soft foil, or, if he did not, the subject was exhaustively treated in every text book upon operative dentistry before the profession, and that all the real, late improvements have been made with adhesive foil, and consequently must be talked and written about, if shared by the profession at large. And we suspect that if the Professor had thought of this he would have been saved the few random shots he aimed in this direction.

This writer throws another sop to those who, instead of industriously striving to excel, are puzzling themselves "how not to do it," when he ridicules the use of the rubber dam. It is useless to multiply words, but we do not envy the man who, after becoming sufficiently familiar with this grand invention to entitle his opinion to any consideration, can speak of it in a way that would lead a beginner to believe it a humbug, and that "a little napkin" was just the thing instead.

His reflections on the practice of describing methods of operating, or publishing figures of patterns of pluggers in the dental periodicals, if not intended to make light of such action, certainly has that tendency, and so it will be felt by those who have so offended. We had supposed that every one who reads these periodicals, or attended these societies, felt benefited by these things. If not, why should there be societies or periodicals? He says we have advanced too far already; that "we need a little retracing of steps." These will be delightful words of cheer to the "rear guard." There always were some who preferred a leveling *down* to a leveling *up*. This "retracing of steps" will put them in the advance by that royal road that we had been taught did not exist—one leading to eminence without labor.

Bring the advance down to them and they will declare it a grand thing. Like Bludsoe in a fight, the Professor has been 'awkward and keerless" of facts; something that may answer with the sensational newspaper writer, but illy becomes a contributor to a scientific journal. In the former, everything is made to do duty to sensation and amusement; in the latter, everything should bend to the truth.

